# Medical Intelligence

**Published:** 1819-10-01

**Authors:** 


### MEDICAL INTELLIGENCE.

---

### *Associated Apothecaries and Surgeon Apothecaries of England and Wales.*

Crown and Anchor Tavern, July 21, 1819.

### ANNUAL GENERAL MEETING.

A Code of Laws for the future government of the Association, drawn up by the Committee, agreeably to the Resolutions of the last Annual General Meeting; and having received a few alterations, was unanimously adopted.

The Chairman then stated, that the Committee extremely regretted their inability to propose any mode of establishing a Board of Examiners of Practitioners in Midwifery, except by the interference of Parliament. The Committee was satisfied that the Society of Apothecaries, by persevering in the manner in which they had hitherto done, in employing the powers they possessed by the Act for regulating the Practice of Apothecaries, would in future, prevent the admission of ignorant and unqualified persons as practitioners in that particular branch of the Profession. But they much regretted, that in consequence of the rejection of the Bill for regulating the Practice of Surgery, the public are still exposed to all the mischiefs which must result from the ignorant and unqualified being allowed to practice both Surgery and Midwifery. It was, however, hoped, that from the communications which the Committee will now receive from the Members of this Association, residing in the country, the statement of such facts will be obtained as may serve to manifest the necessity of Parliament itself interfering, to protect the public from the baneful consequences of being led to trust to ignorant presumption for relief in those situations in which their dearest interests are involved.

A Report of the state of the Funds was then laid before the meeting; viz.

*State of the Funds, July 21, 1819.*

| | | | | | | | |
|---|---|---|---|---|---|---|---|
| To advertisements and sundries for Part of 1817 and 1818 - - - £. | 12 | 0 | 9 | Balance in Bankers Hands £. | 161 | 12 | 9 |
| Mr. Ottey, for Committee Room - - - - - - | 13 | 14 | 0 | By 3 Half-Years' Interest on £. 500 Reduced - | 22 | 10 | 0 |
| Mr. Druce, Solicitor - - | 18 | 7 | 0 | £. | 184 | 2 | 9 |
| Secretary's Salary - - - | 15 | 15 | 0 | | 59 | 16 | 9 |
| £. | 59 | 16 | 9 | Balance in Bankers Hands £. | 124 | 6 | 0 |
| | | | | 3 per Cents. Reduced £. 500 | | | |

The following Resolutions were then carried unanimously:

1. Resolved, That the Rules, &c. now read and approved be those by which the Association shall be in future regulated.

2. Resolved, That the Rules, &c. be printed, and a copy sent to every member of the Association.

3. Resolved, That the thanks of this Association be given to the General Committee for their zeal and ability in preparing the Rules, &c. and for their general attention in forwarding the interests of the Association.

4. Resolved, That the best thanks of this Association are due to the Chairman, James Parkinson, for the great ability and unremitting zeal with which he has, on all occasions, conducted the business of this Association, and that he be requested to continue in the office for the present.

5. Resolved, That the thanks of this Association be given to the Treasurers for their services, and that they be requested to continue in the office for the present.

6. Resolved, That Arthur Tegart, Esq. and Joseph Hayes, Esq. be elected Vice Presidents of this Association.

7. Resolved, That Thomas Graham, Esq. William Hillman, Esq. Lewis Leese, Esq. and George Rodd, Esq. be elected members of the Committee, (Vice) James Parkinson, Esq. President, Joseph Hayes, Esq. elected Vice-President, —Everard Brande, Esq. resigned, and Philip de Bruyn, Esq. deceased.

8. Resolved, That the thanks of this Association be given to the Secretary, John Powell, Esq. for the zeal and ability with which he has fulfilled the duties of his important office.

9. Resolved, That the thanks of this Association be given to Dr. Uwins, the Editor of the London Medical Repository, for his polite attention to the request of this Association, in publishing their Reports in his valuable Journal, and for his liberality to the Association on those occasions.

10. Resolved, That the thanks of this Association be given to Dr. James Johnson, the Editor of the "Medico-Chirurgical Journal, or London Medical Review," for his handsome offer, of the service of his valuable work, for the promulgation of any Papers which may contribute to the objects in view of this Association.

11. Resolved, That Dr. Uwins and Dr. James Johnson be elected *Honorary* Members of this Association.

12. Resolved, That the Editors of the London Medical Repository and of the London Medico-Chirurgical Journal, be requested to oblige this Association by inserting the preceding Resolutions, and state of the Funds, &c. in their respective Publications.

JAMES PARKINSON,
Chairman of the Committee.

---

*At the last Court of the Royal College of Surgeons, the Master and Governors have been changed; the List now stands as follows:*

Master, Sir David Dundas, Bart. Serjeant-Surgeon to the King, Richmond.—Governors, Thomson Forster, Surgeon to Guy's Hospital, Southampton-street; Sir Everard Home, Bart. Surgeon to St. George's Hospital, and Serjeant-Surgeon to the King, Sackville-street.—Court of Assistants, George Chandler, Surgeon to St. Thomas's Hospital, Stamford-street; Thomas Keate, Surgeon to the Prince Regent, Chelsea; John Heaviside, Surgeon Extraordinary to the King, George-street, Hanover-square; Sir William Blizard, Knt. Surgeon to the London Hospital, Devonshire square; Henry Cline, Lincoln's-inn-fields; William Norris, Old Jewry; John Adair Hawkins, Great Marlborough-street: Francis Knight, Saville-row; Sir Ludford Harvey, Kt. Surgeon to St. Bartholomew's Hospital, Red Lion-square; William Lynn, Surgeon to the Westminster Hospital, Parliament-street; John Abernethy, Surgeon to St. Bartholomew's Hospital, Bedford-row; William Lucas, Surgeon to Guy's Hospital, Threadneedle-street; Astley P. Cooper, Surgeon to Guy's Hospital, Spring-gardens; Anthony Carlisle, Surgeon to the Weſtminster Hospital, and Surgeon-Extraordinary to the Prince Regent, Soho-square; Thomas Chevalier, Surgeon-Extraordinary to the Prince Regent, South Audley-street: James Wilson, George-street, Hanover-square; John Gunning, Inspector of Army Hospitals, Surgeon to St. George's Hospital, and Surgeon Extraordinary to the King, Lower Grosvenor-street; Honoratus L. Thomas, Leicester-place.

---

### PLAN
### OF
### A PRACTICAL SCHOOL
### FOR TEACHING
### *OPHTHALMIC SURGERY.*

Sir William Adams having had the honour to be nominated by His Majesty's Government, to superintend the Institution appropriated to the reception of the Blind Pensioners belonging to the army, navy, and artillery, has felt it a duty, to lay open to the profession at large, his improved modes of treating these patients.

With this view he published, in the beginning of March, 1818, a general invitation to the profession to witness his operations and practice upon these pensioners; which invitation has been answered by the attendance of several hundreds of professonal gentlemen, both civil and military.

During the same period Sir William Adams has, from time to time, delivered short courses of Clinical Lectures, in which, after describing the operations and modes of practice hitherto employed in the treatment of the diseases under consideration, he has pointed out their defects, and explained by what means these defects might be obviated.

On fixed days he has performed his operations in the presence of the professional visitors, exhibited to them his modes of treatment, and upon every occasion has particularly directed their attention to the *results*. Those results are now before the public.

This mode of proceeding having obtained the approbation of those who attended at the Government Hospital, and having received numerous applications from the pupils, Sir William Adams now proposes to form a regular School for teaching Ophthalmic Surgery.

The Government Establishment of itself, however, being insufficient for this purpose, (the cases of the pensioners being almost exclusively of the *Chronic* kind,) a Dispensary in its vicinity, for the admission of the poor in civil life, will be immediately established, by which ample opportunity will be afforded of shewing the various *acute* forms of disease.

To render the School complete, Sir William Adams further proposes to deliver Lectures Annually, on the Theory and Treatment of all the important Diseases of the Eye, in which it will be his particular care, whenever a practice differing from the usual routine is recommended, to refer all the points of difference to the test of practical effects, produced under the inspection of the pupils.

Thus the pupil will be afforded a Clinical Course of instruction; added to which he will witness a larger share of operative Practice than in any other Institution of the kind, in this, or probably any other country; it being expected that upwards of two thousand men already pensioned, (the majority of whom require operations,) will undergo treatment in the Government Hospital, independently of the important cases which may apply for relief at the Dispensary.

1st. The Institution about to be established for the reception and treatment of the Poor in Civil life, will, from its local situation, be called the Marylebone Dispensary for Curing Diseases of the Eye.

2d. The poor will receive advice and medicines, two days in each week, viz. on Monday and Friday mornings, from ten to twelve o'clock.

3d. Gentlemen who enter as pupils to the Dispensary, will be permitted to witness the practice and operations in the Hospital founded by Government, for the treatment of the Blind Pensioners.

4th. All important operations will be performed at the Ophthalmic Hospital on Friday mornings, from nine to ten o'clock.

5th. All Medical Officers belonging to the three branches of the Public Service, will be admitted to attend the Practice of both Institutions gratis, upon producing an official recommendation from the heads of their respective departments. This recommendation should be enclosed to SIR WILLIAM ADAMS, and left with the resident Surgeon of the Government Hospital, situate near the Regent's Park.

*Terms of Attendance on the Practice.*

| | | | |
|---|---|---|---|
| Three Months - - - - - - - - - | £.6 | 6 | 0 |
| Six ditto - - - - - - - - - - | 10 | 10 | 0 |
| Twelve ditto - - - - - - - - - | 15 | 15 | 0 |
| Perpetual - - - - - - - - - - | 21 | 0 | 0 |

N.B. A Prospectus of the Lectures will be published a short time previous to their delivery.

---

Mr. CURTIS will commence his next Course of Lectures on the Anatomy, Physiology, and Pathology of the Ear, on Friday October 1st, at the Royal Dispensary for Diseases of the Ear, Carlisle Street.—For particulars apply to Mr. C. at his house, No. 2, Soho Square.—The Royal Dispensary is open to pupils.

Dr. J. B. DAVIS, Physician of the Universal Dispensary for Children, the London Dispensary, and the Surrey Dispensary, will commence his next Course of Lectures on the Diseases and Medicinal Management of Children and Young Persons, at his house, No. 103, Great Surrey Street, Blackfriars, on Wednesday the 13th of October.—Pupils are admitted, as usual, to the practice of the Universal Dispensary for Children, and have the privilege of attending the practice of the London Dispensary without any additional payment.—For particulars apply at the Universal Dispensary for Children, No. 5, St. Andrew's Hill, Doctors' Commons; or at 103, Great Surrey Street, Blackfriars.

We understand that Mr. Parmly, an ingenious Surgeon-Dentist in this metropolis, and the Author of a Practical Work on Diseases of the Teeth, is about to deliver a popular Course of Lectures on that subject; in which he traces a great many constitutional diseases to morbific gases, swallowed or inhaled from carious teeth and foul states of the mouth and gums.

---

Dr. JOUENNE, of Brussels, has committed to the press a Translation into the French, of Dr. Johnson's work on the "Influence of the Atmosphere, &c.;" and he proposes translating the same Author's Work on Tropical Climates afterwards.

LATELY PUBLISHED,

No. I. of the First Series of a Work entitled, Hortus Cryptogamicus Edinensis, being a Collection of the Cryptogamic Tribes of Plants indigenous to Scotland. By J. Stewart, Member of the Wernerian Society, President of the Royal Physical Society, &c. and Lecturer on Botany in Edinburgh.—This first Series is in 8vo, and will embrace Mosses, Hepaticae, Confervae, the smaller Ulvae, and the smaller species of other genera of the submersed Algae.

